# Development and validation of the oral presentation evaluation scale (OPES) for nursing students

**DOI:** 10.1186/s12909-022-03376-w

**Published:** 2022-04-26

**Authors:** Yi-Chien Chiang, Hsiang-Chun Lee, Tsung-Lan Chu, Chia-Ling Wu, Ya-Chu Hsiao

**Affiliations:** 1 Department of Nursing, Chang Gung University of Science and Technology, Division of Pediatric Hematology and Oncology, Linkou Chang Gung Memorial Hospital, Taoyuan City, Taiwan, Republic of China; 2 Department of Nursing, Chang Gung University of Science and Technology, Taoyuan City, Taiwan, Republic of China; 3 Administration Center of Quality Management Department, Chang Gung Medical Foundation, Taoyuan City, Taiwan, Republic of China; 4 Department of Nursing, Chang Gung University of Science and Technology; Administration Center of Quality Management Department, Linkou Chang Gung Memorial Hospital, No.261, Wenhua 1st Rd., Guishan Dist, Taoyuan City, 333 03 Taiwan, Republic of China

**Keywords:** Evaluation, Nurse educators, Nursing students, Oral presentation, Scale development

## Abstract

**Background:**

Oral presentations are an important educational component for nursing students and nursing educators need to provide students with an assessment of presentations as feedback for improving this skill. However, there are no reliable validated tools available for objective evaluations of presentations. We aimed to develop and validate an oral presentation evaluation scale (OPES) for nursing students when learning effective oral presentations skills and could be used by students to self-rate their own performance, and potentially in the future for educators to assess student presentations.

**Methods:**

The self-report OPES was developed using 28 items generated from a review of the literature about oral presentations and with qualitative face-to-face interviews with university oral presentation tutors and nursing students. Evidence for the internal structure of the 28-item scale was conducted with exploratory and confirmatory factor analysis (EFA and CFA, respectively), and internal consistency. Relationships with Personal Report of Communication Apprehension and Self-Perceived Communication Competence to conduct the relationships with other variables evidence.

**Results:**

Nursing students’ (*n* = 325) responses to the scale provided the data for the EFA, which resulted in three factors: accuracy of content, effective communication, and clarity of speech. These factors explained 64.75% of the total variance. Eight items were dropped from the original item pool. The Cronbach’s α value was .94 for the total scale and ranged from .84 to .93 for the three factors. The internal structure evidence was examined with CFA using data from a second group of 325 students, and an additional five items were deleted. Except for the adjusted goodness of fit, fit indices of the model were acceptable, which was below the minimum criteria. The final 15-item OPES was significantly correlated with the students’ scores for the Personal Report of Communication Apprehension scale (*r* = −.51, *p* < .001) and Self-Perceived Communication Competence Scale (*r* = .45, *p* < .001), indicating excellent evidence of the relationships to other variables with other self-report assessments of communication.

**Conclusions:**

The OPES could be adopted as a self-assessment instrument for nursing students when learning oral presentation skills. Further studies are needed to determine if the OPES is a valid instrument for nursing educators’ objective evaluations of student presentations across nursing programs.

**Supplementary Information:**

The online version contains supplementary material available at 10.1186/s12909-022-03376-w.

## Background

Competence in oral presentations is important for medical professionals to communicate an idea to others, including those in the nursing professions. Delivering concise oral presentations is a useful and necessary skill for nurses [1, 2]. Strong oral presentation skills not only impact the quality of nurse-client communications and the effectiveness of teamwork among groups of healthcare professionals, but also promotion, leadership, and professional development [2]. Nurses are also responsible for delivering health-related knowledge to patients and the community. Therefore, one important part of the curriculum for nursing students is the delivery of oral presentations related to healthcare issues. A self-assessment instrument for oral presentations could provide students with insight into what skills need improvement.

Three components have been identified as important for improving communication. First, a presenter’s self-esteem can influence the physio-psychological reaction towards the presentation; presenters with low self-esteem experience greater levels of anxiety during presentations [3]. Therefore, increasing a student’s self-efficacy can increase confidence in their ability to effectively communicate, which can reduce anxiety [3, 4]. Second, Liao (2014) reported improving speaking efficacy can also improve oral communications and collaborative learning among students could improve speech efficacy and decrease speech anxiety [5]. A study by De Grez et al. provided students with a list of skills to practice, which allowed them to feel more comfortable when a formal presentation was required, increased presentation skills, and improved communication by improving self-regulation [6]. Third, Carlson and Smith-Howell (1995) determined quality and accuracy of the information presented was also an important aspect of public speaking performances [7]. Therefore, all three above mentioned components are important skills for effective communication during an oral presentation.

Instruments that provide an assessment of a public speaking performance are critical for helping students’ improve oral presentation skills [7]. One study found peer evaluations were higher than those of university tutors for student presentations, using a student-developed assessment form [8]. The assessment criteria included content (40%), presentation (40%), and structure (20%); the maximum percent in each domain was given for “excellence”, which was relative to a minimum “threshold”. Multiple “excellence” and “threshold” benchmarks were described for each domain. For example, benchmarks included the use of clear and appropriate language, enthusiasm, and keeping the audience interested. However, the percentage score did not provide any information about what specific benchmarks were met. Thus, these quantitative scores did not include feedback on specific criteria that could enhance future presentations.

At the other extreme is an assessment that is limited to one aspect of the presentation and is too detailed to evaluate the performance efficiently. An example of this is the 40-item tool developed by Tsang (2018) [6] to evaluate oral presentation skills, which measured several domains: voice (volume and speed), facial expressions, passion, and control of time. An assessment tool developed by De Grez et al. (2009) includes several domains: three subcategories for content (quality of introduction, structure, and conclusion), five subcategories of expression (eye-contact, vocal delivery, enthusiasm, interaction with audience, and body-language), and a general quality [9]. Many items overlap, making it hard to distinguish specific qualities. Other evaluation tools include criteria that are difficult to objectively measure, such as body language, eye-contact, and interactions with the audience [10]. Finally, most of the previous tools were developed without testing the reliability and validity of the instrument.

Nurses have the responsibility of not only providing medical care, but also medical information to other healthcare professionals, patients, and members of the community. Therefore, improving nursing students’ speaking skills is an important part of the curriculum. A self-report instrument for measuring nursing students’ subjective assessment of their presentation skills could help increase competence in oral communication. However, to date, there is a no reliable and valid instrument of evaluating oral presentation performance in nursing education. Therefore, the aim of this study was to develop a self-assessment instrument for nursing students that could guide them in understanding their strengths and development areas in aspects of oral presentations. Development of a scale that is a valid and reliable instrument for nursing students could then be examined for use as a scale for objective evaluations of oral presentations by peers and nurse educators.

## Methods

### Study design

This study developed and validated an oral presentation evaluation scale (OPES) that could be employed as a self-assessment instrument for students when learning skills for effective oral presentations. The instrument was developed in two phases: Phase I (item generation and revision) and Phase II (scale development) [11]. The phase I was aimed to generate items by a qualitative method and to collect content evidence for the OPES. The phase II focused on scale development which was established internal structure evidence including CFA, EFA, and internal consistency of the scale for the OPES. In addition, the phase II collected the evidence of OPES on relationships with other variables. Because we hope to also use the instrument as an aid for nurse educators in objective evaluations of nursing students’ oral presentations, both students and educators were involved in item generation and revision. Only nursing students participated in Phase II.

Approval was obtained from Chang Gung Medical Foundation institutional review board (ID: 201702148B0) prior to initiation of the study. Informed consent was obtained from all participants prior to data collection. All participants being interviewed for item generation in phase I provided signed informed consent indicating willingness to be audiotaped during the interview. All the study methods were carried out in accordance with relevant guidelines and regulations.

### Phase I: item generation and item revision

#### Participants

A sample of nurse educators (*n* = 8) and nursing students *(n* = 11) participated in the interviews for item generation. Nursing students give oral presentations to meet the curriculum requirement, therefore the educators were university tutors experienced in coaching nursing students preparing to give an oral presentation. Nurse educators specializing in various areas of nursing, such as acute care, psychology, and community care were recruited if they had at least 10 years’ experience coaching university students. The mean age of the educators was 52.1 years (*SD* = 4.26), 75% were female, and the mean amount of teaching experience was 22.6 years (*SD* = 4.07). Students were included if they had given at least one oral presentation and were willing to share their experiences of oral presentation. The mean age of the students was 20.7 (*SD* = 1.90), 81.8% were female; 36.3%, four were second year students, three were third students, and four were in their fourth year.

An additional eight educators participated in the evaluation of content evidence of the ORES. All had over 10 years’ experience in coaching students in giving an oral presentation that would be evaluated for a grade.

#### Item generation

Development of item domains involved deductive evaluations of the about oral presentations [2, 3, 6–8, 12–14]. Three domains were determined to be important components of an oral presentation: accuracy of content, effective communication, and clarity of speech. Inductive qualitative data from face-to-face semi-structured interviews with nurse educators and nursing student participants were used to identify domain items [11]. Details of interview participants are described in the section above. The interviews with nurse educators and students followed an interview guide (Table 1) and lasted approximately 30–50 min for educators and 20–30 min for students. Deduction of the literature and induction of the interview data was used to determine categories considered important for the objective evaluation of oral presentations.

**Table 1:** Interview guide for semi-structured interviews with nurse educators and nursing students for item generation

Participant group	Questions
Educator	1.What has been your reaction to oral reports or presentations given by your students?
	2. What problems commonly occur when students are giving oral reports or presentations?
	3. In your opinion, what do you consider a good presentation, and could you describe the characteristics?
	4. How do you evaluate the performance of the student’s oral reports or presentations? Are there any difficulties or problems evaluating the oral reports?
Student	1. Would you please tell me about your experiences of giving an oral report or presentation?
	2. In your opinion, what is a good presentation and what are some of the important characteristics?

*Analysis of interview data.* Audio recordings of the interviews were transcribed verbatim at the conclusion of each interview. Interview data were analyzed by the first, second, and corresponding author, all experts in qualitative studies. The first and second authors coded the interview data to identify items educators and student described as being important to the experience of an oral presentation [11]. The corresponding author grouped the coded items into constructs important for oral presentations. Meetings with the three researchers were conducted to discuss the findings; if there were differences in interpretation, an outside expert in qualitative studies was included in the discussions until consensus was reached among the three researchers.

Analysis of the interview data indicated items involved in preparation, presentation, and post-presentation were important to the three domains of accuracy of content, effective communication, and clarity of speech. Items for accuracy of content involved preparation (being well-prepared before the presentation; preparing materials suitable for the target audience; practicing the presentation in advance) and post-presentation reflection; and discussing the content of the presentation with classmates and teachers. Items for effective communication involved the presentation itself: obtain the attention of the audience; provide materials that are reliable and valuable; express confidence and enthusiasm; interact with the audience; and respond to questions from the audience. The third domain, clarity of speech, involved of items could be, post-presentation, involved a student’s ability to reflect on the content and performance of their presentation and willingness to obtain feedback from peers and teachers.

#### Item revision: content evidence

Based on themes that emerged during, 28 items were generated. Content evidence of the 28 items of the OPES was established with a panel of eight experts who were educators that had not participated in the face-to-face interviews. The experts were provided with a description of the research purpose, a list of the proposed items, and were asked to rate each item on a 4-point Likert scale (1 = not representative, 2 = item needs major revision, 3 = representative but needs minor revision, 4 = representative). For item-level content validity index (I-CVI) was determined by the total items rated 3 or 4 divided by the total number of experts; scale-level content validity index (S-CVI) was determined by the total items rated 3 or 4 divided by the total number of items.

Based on the suggestions of the experts, six items of the OPES were reworded for clarity: item 12 was revised from “The presentation is riveting” to “The presenter’s performance is brilliant; it resonates with the audience and arouses their interests”. Two items were deleted because they duplicated other items: “demonstrates confidence” and “presents enthusiasm” were combined and item 22 became, “demonstrates confidence and enthusiasm properly”. The item “the presentation allows for proper timing and sequencing” and “the length of time of the presentation is well controlled” were also combined into item 9, “The content of presentation follows the rules, allowing for the proper timing and sequence”. Thus, a total of 26 items were included in the OPES at this phase. The I-CVI value was .88 ~ 1 and the scale-level CVI/universal agreement was .75, indicating that the OPES was an acceptable instrument for measuring an oral presentation [11].

### Phase II: scale development

Phase II, scale development, aimed to establish the internal structure evidence for OPES. The evidence of relation to other variables was also evaluated as well in this phase. More specifically, the internal structure evidence for OPES was evaluated by exploratory factor analysis (EFA) and confirmatory factor analysis (CFA). The evidence of relationships to other variables was determined by examining the relationships between the OPES and the PRCA and SPCC [15].

#### Participants

A sample of nursing students was recruited purposively from a university in Taiwan. Students were included if they were: (a) full-time students; (b) had declared nursing as their major; and (c) were in their sophomore, junior, or senior year. First-year university students (freshman) were excluded. A bulletin about the survey study was posted outside of classrooms; 707 students attend these classes. The bulletin included a description of the inclusion criteria and instructions to appear at the classroom on a given day and time if students were interested in participating in the study. Students who appeared at the classroom on the scheduled day (*N* = 650) were given a packet containing a demographic questionnaire (age, gender, year in school), a consent form, the OPES instrument, and two scales for measuring aspects of communication, the Personal Report of Communication Apprehension (PRCA) and the Self-Perceived Communication Competence (SPCC); the documents were labeled with an identification number to anonymize the data. The 650 students were divided into two groups, based on the demographic data using the SPSS random case selection procedure, (Version 23.0; SPSS Inc., Chicago, IL, USA). The selection procedure was performed repeatedly until the homogeneity of the baseline characteristics was established between the two groups (*p* > .05). The mean age of the participants was 20.5 years (*SD* = 0.98) and 87.1% were female (*n* = 566). Participants were comprised of third-year students (40.6%, *n* = 274), fourth year (37.9%, *n* = 246) and second year (21.5%, *n* = 93). The survey data for half the group (calibration sample, *n* = 325) was used for EFA; the survey data from the other half (the validation sample, *n* = 325) was used for CFA. Scores from the PRCA and SPCC instruments were used for evaluating the evidence of relationships to other variables.

The aims of phase II were to collect the scale of internal structure evidence, which identify the items that nursing students perceived as important during an oral presentation and to determine the domains that fit a set of items. The 325 nursing students for EFA (described above) were completed the data collection. We used EFA to evaluate the internal structure of the scale. The items were presented in random order and were not nested according to constructs. Internal consistency of the scale was determined by calculating Cronbach’s alpha.

Then, the next step involved determining if the newly developed OPES was a reliable and valid self-report scale for subjective assessments of nursing students’ previous oral presentations. Participants (the second group of 325 students) were asked, “How often do you incorporate each item into your oral presentations?”. Responses were scored on a 5-point Likert scale with 1 = never to 5 = always; higher scores indicated a better performance. The latent structure of the scale was examined with CFA.

Finally, the evidence of relationships with other variables of the OPES was determined by examining the relationships between the OPES and the PRCA and SPCC, described below.

#### The 24-item PRCA scale

The PRCA scale is a self-report instrument for measuring communication apprehension, which is an individual’s level of fear or anxiety associated with either real or anticipated communication with a person or persons [12]. The 24 scale items are comprised of statements concerning feelings about communicating with others. Four subscales are used for different situations: group discussions, interpersonal communications, meetings, and public speaking. Each item is scored on a 5-point Likert scale from 1 (strongly disagree) to 5 (strongly agree); scores range from 24 to 120, with higher scores indicating greater communication anxiety. The PRCA has been demonstrated to be a reliable and valid scale across a wide range of related studies [5, 13, 14, 16, 17]. The Cronbach’s alpha for the scale is .90 [18]. We received permission from the owner of the copyright to translate the scale into Chinese. Translation of the scale into Chinese by a member of the research team who was fluent in English was followed by back-translation from a differed bi-lingual member of the team to ensure semantic validity of the translated PRCA scale. The Cronbach’s alpha value in the present study was .93.

#### The 12-item SPCC scale

The SPCC scale evaluates a persons’ self-perceived competence in a variety of communication contexts and with a variety of types of receivers. Each item is a situation which requires communication, such as “Present a talk to a group of strangers”, or “Talk with a friend”. Participants respond to each situation by ranking their level of competence from 0 (completely incompetent) to 100 (completely competent). The Cronbach’s alpha for reliability of the scale is .85. The SPCC has been used in similar studies [13, 19]. We received permission owner of the copyright to translate the scale into Chinese. Translation of the SPCC scale into Chinese by a member of the research team who was fluent in English was followed by back-translation from a differed bi-lingual member of the team to ensure semantic validity of the translated scale. The Cronbach’s alpha value in the present study was .941.

### Statistical analysis

Data were analyzed using SPSS for Windows 23 (SPSS Inc., Chicago, IL, USA). Data from the 325 students designated for EFA was used to determine the internal structure evidence of the OPES. The Kaiser-Meyer-Olkin measure for sampling adequacy and Bartlett’s test of sphericity demonstrated factor analysis was appropriate [20]. Principal component analysis (PCA) was performed on the 26 items to extract the major contributing factors; varimax rotation determined relationships between the items and contributing factors. Factors with an eigenvalue > 1 were further inspected. A factor loading greater than .50 was regarded as significantly relevant [21].

All item deletions were incorporated one by one, and the EFA model was respecified after each deletion, which reduced the number of items in accordance with a priori criteria. In the EFA phase, the internal consistency of each construct was examined using Cronbach’s alpha, with a value of .70 or higher considered acceptable [22].

Data from the 325 students designated for CFA was used to validate the factor structure of the OPES. In this phase, items with a factor loading less than .50 were deleted [21]. The goodness of the model fit was assessed using the following: absolute fit indices, including goodness of fit index (GFI), adjusted goodness of fit index (AGFI), standardized root mean squared residual (SRMR), and the root mean square error of approximation (RMSEA); relative fit indices, normed and non-normed fit index (NFI and NNFI, respectively), and comparative fit index (CFI); and the parsimony NFI, CFI, and likelihood ratio (*x*^*2*^*/df*) [23].

In addition to the validity testing, a research team, which included a statistician, determined the appropriateness of either deleting or retaining each item. The convergent validity (internal quality of the items and factor structures), was further verified using standardized factor loading, with values of .50 or higher considered acceptable, and average variance extraction (AVE), with values of .5 or higher considered acceptable [21]. Convergent reliability (CR) was assessed using the construct reliability from the CFA, with values of .7 or higher considered acceptable [24]. The AVE and correlation matrices among the latent constructs were used to establish discriminant validity of the instrument. The square root of the AVE of each construct was required to reach a value that was larger than the correlation coefficient between itself and the other constructs [24].

The evidence of relationships with other variables was determined by examining the relationship of nursing students’ scores (*N* = 650) on the newly developed OPES with scores for constructs of communication of the translated scales for PRCA and SPCC. The hypotheses between OPES to PRCA and SPCC individually indicated the strong self-reported presentation competence were associated with lower communication anxiety and greater communication competence.

## Results

### Development of the OPES: internal structure evidence

EFA was performed sequentially six times until there were no items with a loading factor < .50 or that were cross-loaded, and six items were deleted (Table 2). EFA resulted in 20 items with a three factors solution, which represented 64.75% of the variance of the OPES. The Cronbach’s alpha estimates for the total scale was .94. indicating the scale had sound internal reliability (Table 2). The three factors were labeled in accordance with the item content via a panel discussion and had Cronbach’s alpha values of .93, .89, and .84 for factors 1, 2 and 3, respectively.

**Table 2:** Summary of exploratory factor analysis: descriptive statistics, factor loading, and reliability for nursing students (*N* = 325)

		Score		Factor loading		
Item	Description	Mean	SD	1	2	3
7	The content of the presentation matches the theme	4.25	0.62	.76	.20	.17
14	Presentation aids, such as PowerPoint and posters, highlight key points of the report	4.21	0.74	.75	.21	.30
15	Proper use of presentation aids such as PowerPoint and posters	4.32	0.69	.74	.12	.28
8	The content of the presentation is clear and focused	4.02	0.69	.72	.36	.11
10	The content of the presentation is organized and logical	3.93	0.75	.72	.38	.13
4	Preparation of presentation aids, such as PowerPoint and posters, in advance	4.53	.67	.70	−.10	.20
16	Presentation aids, such as PowerPoint and posters, help the audience understand the content of the presentation	4.26	0.68	.69	.20	.37
9	The organization of the presentation is structured to provide the necessary information, while also adhering to time limitations	4.10	0.69	.68	.30	.18
11	The content of the presentation provides correct information	4.12	0.66	.68	.31	.10
1	Preparation of the content in accordance with the theme and rules in advance	4.49	0.61	.64	−.02	.39
13	The entire content of the presentation is prepared in a way that is understandable to the audience	3.99	0.77	.61	.40	.09
22	Presenter demonstrates confidence and an appropriate level of enthusiasm	3.92	0.91	.17	.83	.25
21	Presenter uses body language in a manner that increases the audience’s interest in learning	3.50	0.95	.09	.81	.22
24	Presenter interacts with the audience using eye contact during the question and answer session	3.65	0.92	.15	.77	.24
23	Presenter responds to the audience’s questions properly	3.63	0.87	.23	.77	.17
12	The presenter’s performance is brilliant; it resonates with the audience and arouses their interests	3.43	0.78	.43	.65	.04
17	The pronunciation of the words in the presentation is correct	3.98	0.82	.31	.29	.74
18	The tone and volume of the presenter’s voice is appropriate	3.82	0.82	.22	.50	.70
19	The words and phrases of the presenter are smooth and fluent	3.70	0.82	.26	.52	.65
20	The clothing worn by the presenter is appropriate	4.16	0.77	.33	.12	.57
	Eigenvalue (sum of squared loading)			6.01	4.34	2.60
	Explained variance			30.03%	21.72%	13.00%
	Cumulative variance			30.03%	51.75%	64.75%
	Cronbach’s α for each subscale			.93	.89	.84
	Cronbach’s α for the total scale			.94		
Item	Deleted following EFA					
2	Considers the background or needs of the audience to prepare the content of the presentation in advance	3.94	0.84			
3	Discusses the content of the presentation with experts, teachers or peers (classmates) in advance	3.94	0.89			
5	Practices several times in private in before the presentation	3.96	0.89			
6	Invites classmates or teachers to watch a rehearsal before the presentation	3.39	1.04			
25	Reflects on the experience as well as the strengths and weaknesses of the presentation	3.83	0.85			
26	Obtains feedback from peers (e.g. classmates), teachers, or an audience	3.92	0.81			

*Abbreviations*:*SD* standard deviation, *EFA* exploratory factor analysis

Factor 1, Accuracy of Content, was comprised of 11 items and explained 30.03% of the variance. Items in *Accuracy of Content* evaluated agreement between the topic (theme) and content of the presentation, use of presentation aids to highlight the key points of the presentation, and adherence to time limitations. These items included statements such as: “The content of the presentation matches the theme” (item 7), “Presentation aids, such as PowerPoint and posters, highlight key points of the report” (item 14), and “The organization of the presentation is structured to provide the necessary information, while also adhering to time limitations” (item 9). Factor 2, “Effective Communication”, was comprised of five items, which explained 21.72% of the total variance. *Effective Communication* evaluated the attitude and expression of the presenter. Statements included “Demonstrates confidence and an appropriate level of enthusiasm” (item 22), “Uses body language in a manner that increases the audience’s interest in learning” (item 21), and “Interacts with the audience using eye contact and a question and answer session” (item 24). Factor 3, “Clarity of Speech” was comprised of four items, which explained 13.00% of the total variance. Factor 3 evaluated the presenter’s pronunciation with statements such as “The words and phrases of the presenter are smooth and fluent” (item 19).

The factor structure of the 20-items of the EFA were examined with CFA. We sequentially removed items 1, 4, 20, 15, and 16, based on modification indices. The resultant 15-item scale had acceptable fit indices for the 3-factor model of the OPES for chi-square (*x*^*2*^*/df* = 2.851), RMSEA (.076), NNFI (.933), and CFI = .945. However, the AGFI, which was .876, was below the acceptable criteria of .9. A panel discussion with the researchers determined that items 4, 15, and 16 were similar in meaning to item 14; item 1 was similar in meaning to item 7. Therefore, the panel accepted the results of the modified CFA model of the OPES with 15 items and 3-factors.

**Fig. 1:**
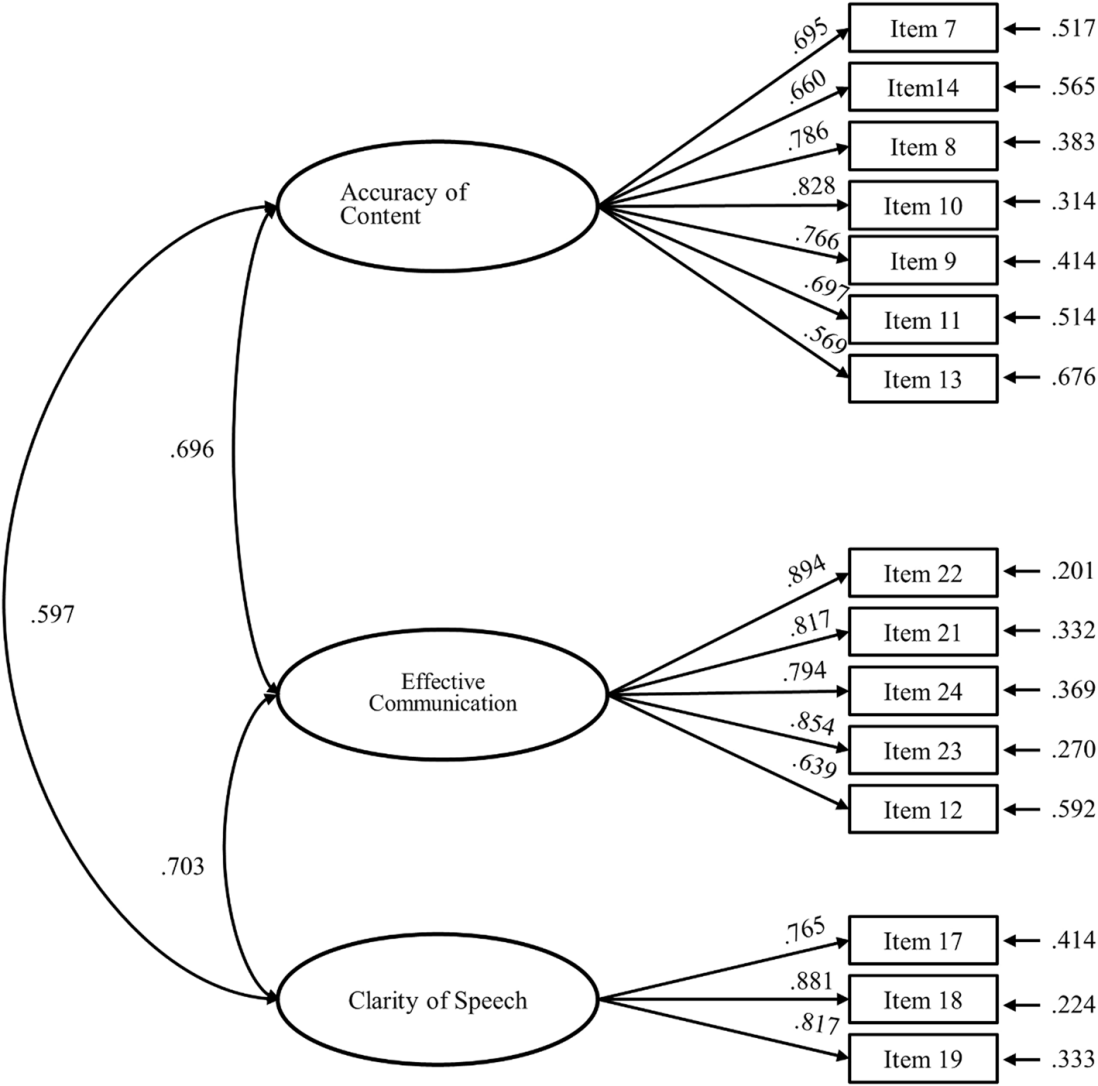
The standardized estimates of CFA model for validation sample

As illustrated in Table 3 and Fig. 1, all standardized factor loadings exceeded the threshold of .50, and the AVE for each construct ranged from .517 to .676, indicating acceptable convergent validity. In addition, the CR was greater than .70 for the three constructs (range = .862 to .901), providing further evidence for the reliability of the instrument [25]. As shown in Table 4, all square roots of the AVE for each construct (values in the diagonal elements) were greater than the corresponding inter-construct correlations (values below the diagonal) [24, 25]. These findings provide further support for the validity of the OPES.

**Table 3:** Confirmatory factor analysis: convergent reliability and validity of the OPES scale for nursing students (*n* = 325)

Construct/Item	Item score		Factor loading		Reliability		
	Mean	*SD*	λ	*t*	*R*^2^	CR	AVE
Accuracy of content						.881	.517
Item 7	4.25	0.60	.695	13.774***	.483		
Item 14	4.23	0.68	.660	12.863***	.435		
Item 8	3.98	0.66	.786	16.352***	.617		
Item 10	3.88	0.69	.828	17.703***	.686		
Item 9	4.03	0.72	.766	15.753***	.586		
Item 11	4.08	0.65	.697	13.835***	.486		
Item 13	3.92	0.78	.569	10.687***	.324		
Effective Communication						.901	.647
Item 22	3.58	0.91	.894	20.230***	.799		
Item 21	3.43	0.97	.817	17.548***	.668		
Item 24	3.69	0.91	.794	16.816***	.631		
Item 23	3.64	0.87	.854	18.802***	.730		
Item 12	3.41	0.79	.639	12.490***	.408		
Clarity of speech						.862	.676
Item 17	3.94	0.76	.765	15.541***	.586		
Item 18	3.81	0.79	.881	19.002***	.776		
Item 19	3.70	0.76	.817	17.026***	.667		

*Note*. *λ* standardized factor loading, *R*^2^ reliability of item (squared multiple correlation, SMC), *CR* construct (component/composite) reliability, *AVE* average variance extraction

*** *p* < .001

**Table 4:** Correlations among the latent variables from confirmatory factor analysis of the OPES scale for nursing students (*n* = 325)

Construct	1	2	3
1. Accuracy of content	.719^a^		
2. Effective communication	.696***	.804^a^	
3. Clarity of speech	.597***	.703***	.822^a^

^a^The value in the diagonal element is the square root of AVE of each construct

***p < .001

### Development of the OPES: relationships with other variables

Relationships with other variable evidence was examined with correlation coefficients for the total score and subscale scores of the OPES with the total score and subscale scores of the PRCA and SPCC (Table 5) from all nursing students who participated in the study and complete all three scales (*N* = 650). Correlation coefficients for the total score of the OPES with total scores for the PRCA and SPCC were − .51 and .45, respectively (both *p* < .001). Correlation coefficients for subscale scores of the OPES with the subscale scores of the PRCA and SPCC were all significant (*p* < .001), indicating strong valid evidence of the scale as a self-assessment for effective communication.

**Table 5:** Correlation coefficients for total scores and subscale scores for the OPES, PRCA, and SPCC

Instruments & subscales	1	2	3	4	5	6	7	8	9	10	11	12	13	14	15	16
1. OPES																
2. Accuracy of content	**.82**															
3. Effective Communication	**.89**	**.62**														
4. Clarity of speech	**.87**	**.58**	**.64**													
5. PRCA	**−.51**	**−.38**	**−.53**	**−.39**												
6. Group discussion	**−.35**	**−.30**	**−.36**	**−.25**	**.73**											
7. Meetings	**−.46**	**−.33**	**−.48**	**−.35**	**.90**	**.57**										
8. Interpersonal	**−.41**	**−.30**	**−.42**	**−.32**	**.86**	**.54**	**.69**									
9. Public Speaking	**−.48**	**−.32**	**−.51**	**−.39**	**.84**	**.40**	**.71**	**.64**								
10. SPCC	**.45**	**.35**	**.46**	**.34**	**−.55**	**−.35**	**−.46**	**−.53**	**−.50**							
11. Public	**.48**	**.37**	**.49**	**.36**	**−.57**	**−.35**	**−.50**	**−.49**	**−.55**	**.92**						
12. Meeting	**.41**	**.30**	**.44**	**.30**	**−.53**	**−.33**	**−.45**	**−.52**	**−.48**	**.95**	**.84**					
13. Group	**.41**	**.33**	**.40**	**.32**	**−.49**	**−.33**	**−.40**	**−.48**	**−.43**	**.94**	**.83**	**.86**				
14. Dyad	**.40**	**.33**	**.40**	**.29**	**−.48**	**−.29**	**−.38**	**−.50**	**−.42**	**.94**	**.79**	**.87**	**.88**			
15. Stranger	**.40**	**.30**	**.44**	**.27**	**−.52**	**−.26**	**−.44**	**−.53**	**−.49**	**.93**	**.83**	**.89**	**.87**	**.90**		
16. Acquaintance	**.42**	**.30**	**.43**	**.32**	**−.51**	**−.32**	**−.41**	**−.49**	**−.46**	**.94**	**.88**	**.90**	**.89**	**.87**	**.82**	
17. Friend	**.41**	**.37**	**.37**	**.34**	**−.47**	**−.39**	**−.39**	**−.40**	**−.39**	**.82**	**.79**	**.77**	**.79**	**.75**	**.61**	**.73**

*OPES* Oral Presentation Evaluation Scale, *PRCA* Personal Report of Communication Apprehension, *SPCC* Self-Perceived Communication Competence

Bold figures all *p* < .001.

## Discussion

The 15-item OPES was found to be a reliable and valid instrument for nursing students’ self-assessments of their performance during previous oral presentations. The strength of this study is that the initial items were developed using both literature review and interviews with nurse educators, who were university tutors in oral presentation skills, as well as nursing students at different stages of the educational process. Another strength of this study is the multiple methods used to establish the validity and reliability of the OPES, including internal structure evidence (both EFA and CFA) and relationships with other variables [15, 26].

Similar to previous to other oral presentation instruments, content analysis of items of the OPES generated from the interviews with educators and students indicated accuracy of the content of a presentation and effective communication were important factors for a good performance [3–6, 8]. Other studies have also included self-esteem as a factor that can influence the impact of an oral presentation [3], however, the subscale of effective communication included the item “Demonstrates confidence and an appropriate level of enthusiasm”, which a quality of self-esteem. The third domain was identified as clarity of speech, which is unique to our study.

Constructs that focus on a person’s ability to deliver accurate content are important components for evaluations of classroom speaking because they have been shown to be fundamental elements of public speaking ([7]). Accuracy of content as it applies to oral presentation for nurses is important not only for communicating information involving healthcare education for patients, but also for communicating with team members providing medical care in a clinical setting.

The two other factors identified in the OPES, effective communication and clarity of speech, are similar to constructs for delivery of a presentation, which include interacting with the audience through body-language, eye-contact, and question and answer sessions. These behaviors indicate the presenter is confident and enthusiastic, which engages and captures the attention of an audience. It seems logical that the voice, pronunciation, and fluency of speech were not independent factors because the presenter’s voice qualities all are keys to effectively delivering a presentation. A clear and correct pronunciation, appropriate tone and volume of a presentation assists audiences in more easily receiving and understanding the content.

Our 15-item OPES scale evaluated the performance based on outcome. The original scale was composed of 26 items that were derived from qualitative interviews with nursing students and university tutors in oral presentations. These items were the result of asking about important qualities at three timepoints of a presentation: before, during, and after. However, most of the items that were deleted were those about the period before the presentation (1 to 6); two items (25 and 26) were about the period after the presentation. Analysis did not reflect the qualitative interview data expressed by educators and students regarding the importance of preparing with practice and rehearsal, and the importance of peer and teacher evaluations. Other studies have suggested that preparation and self-reflection is important for a good presentation, which includes awareness of the audience receiving the presentation, meeting the needs of the audience, defining the purpose of the presentation, use of appropriate technology to augment information, and repeated practices to reduce anxiety [2, 5, 27]. However, these items were deleted in the scale validation stage, possibly because it is not possible to objectively evaluate how much time and effort the presenter has devoted to the oral presentation.

The deletion of item 20, “The clothing worn by the presenter is appropriate” was also not surprising. During the interviews, educators and students expressed different opinions about the importance of clothing for a presentation. Many of the educators believed the presenter should be dressed formally; students believed the presenter should be neatly dressed. These two perspectives might reflect generational differences. However, these results are reminders assessments should be based on a structured and objective scale, rather than one’s personal attitude and stereotype of what should be important about an oral presentation.

The application of the OPES may be useful not only for educators but also for students. The OPES could be used a checklist to help students determine how well their presentation matches the 15 items, which could draw attention to deficiencies in their speech before the presentation is given. Once the presentation has been given, the OPES could be used as a self-evaluation form, which could help them make modifications to improve the next the next presentation. Educators could use the OPES to evaluate a performance during tutoring sessions with students, which could help identify specific areas needing improvement prior to the oral presentation. Although, analysis of the scale was based on data from nursing students, additional assessments with other populations of healthcare students should be conducted to determine if the OPES is applicable for evaluating oral presentations for students in general.

### Limitations

This study had several limitations. Participants were selected by non-random sampling, therefore, additional studies with nursing students from other nursing schools would strengthen the validity and reliability of the scale. In addition, the OPES was developed using empirical data, rather than basing it on a theoretical framework, such as anxiety and public speaking. Therefore, the validity of the OPES for use in other types of student populations or cultures that differ significantly from our sample population should be established in future studies. Finally, the OPES was in the study was examined as a self-assessment instrument for nursing students who rated themselves based on their perceived abilities previous oral presentations rather than from peer or nurse educator evaluations. Therefore, applicability of the scale as an assessment instrument for educators providing an objective score of nursing students’ real-life oral presentations needs to be validated in future studies.

## Conclusion

This newly developed 15-item OPES is the first report of a valid self-assessment instrument for providing nursing students with feedback about whether necessary targets for a successful oral presentation are reached. Therefore, it could be adopted as a self-assessment instrument for nursing students when learning what oral presentation require skills require strengthening. However, further studies are needed to determine if the OPES is a valid instrument for use by student peers or nursing educators evaluating student presentations across nursing programs.

## Supplementary Information

**Additional file 1.**

## Data Availability

The datasets and materials of this study are available to the corresponding author on request.
