# Supplementary material for: Development and validation of the oral presentation evaluation scale (OPES) for nursing students
Source: BMC Med Educ. 2022 Apr 26;22:318. doi: 10.1186/s12909-022-03376-w (PMC9040219; doi:10.1186/s12909-022-03376-w)
Supplement: Supplementary file 1 — Additional file 1. [file 12909_2022_3376_MOESM1_ESM.docx]

**Oral presentation performance**

Please check the proper boxes in table area indicated by the thick border below according to your **past** experiences of oral reports (presentations).

Never: Never do this behavior or have never done it before, about less than 10% (0-10%) of chances.

Occasionally: Occasionally do this behavior, about 30% (11-30%) of chances.

Sometimes: About 50% (31-60%) of chances to achieve.

Often: About 70% (61-80%) of chances to achieve.

Always: About 90% (81-100%) of chances to achieve.

| No. | Item | Never | Occasionally | sometimes | usually | always |
| --- | --- | --- | --- | --- | --- | --- |
|  | The content of the presentation matches the theme |  |  |  |  |  |
|  | Presentation aids (e.g. PPT, posters, etc.), highlight the key points of the report |  |  |  |  |  |
|  | The content of presentation is clear and focused |  |  |  |  |  |
|  | The content of presentation is organized and logical |  |  |  |  |  |
|  | The content of presentation follows the rules and allow for proper timing and sequencing. |  |  |  |  |  |
|  | The content of presentation provides correct information |  |  |  |  |  |
|  | Be familiar with the entire content of the presentation. |  |  |  |  |  |
|  | Demonstrating confidence and enthusiasm properly. |  |  |  |  |  |
|  | Using body language properly to increase the learning interest of audiences |  |  |  |  |  |
|  | Interacting with the audiences includes using eye contact and a question and answer session |  |  |  |  |  |
|  | Responding to audiences’ questions properly. |  |  |  |  |  |
|  | The content of presentation is brilliant, and be able to arouse the interests and resonances from audiences |  |  |  |  |  |
|  | The pronunciation of presentation is correct. |  |  |  |  |  |
|  | The tone and volume of presentation are appropriate. |  |  |  |  |  |
|  | The words and phrases of the presenter are smooth and fluent. |  |  |  |  |  |
